# Drug Binding to BamA Targets Its Lateral Gate

**DOI:** 10.1021/acs.jpcb.3c04501

**Published:** 2023-08-17

**Authors:** Katie M. Kuo, Jinchan Liu, Anna Pavlova, James C. Gumbart

**Affiliations:** † School of Chemistry and Biochemistry, Georgia Institute of Technology, Atlanta, Georgia 30332, United States; ‡ Department of Molecular Biophysics and Biochemistry (MB&B), Yale University, New Haven, Connecticut 06510, United States; § School of Physics, Georgia Institute of Technology, Atlanta, Georgia 30332, United States

## Abstract

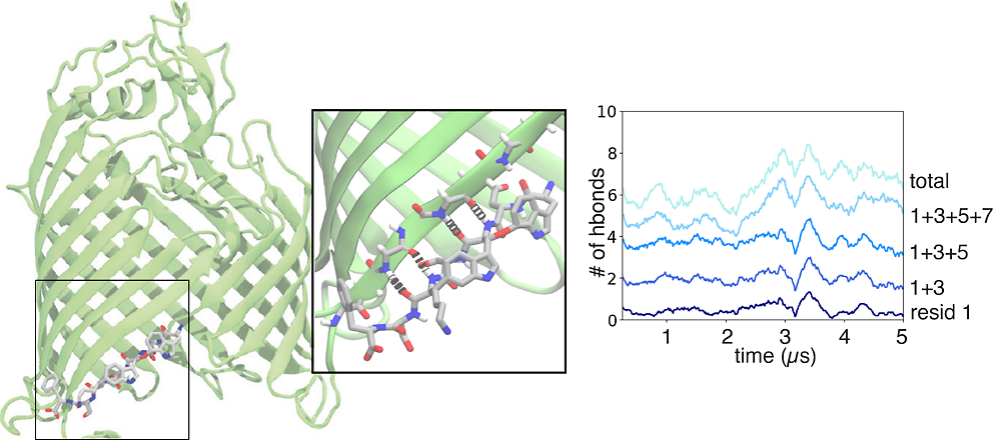

BamA, the core component of the β-barrel assembly machinery (BAM) complex, is an outer-membrane protein (OMP) in Gram-negative bacteria. Its function is to insert and fold substrate OMPs into the outer membrane (OM). Evidence suggests that BamA follows the asymmetric hybrid-barrel model where the first and last strands of BamA separate, a process known as lateral gate opening, to allow nascent substrate OMP β-strands to sequentially insert and fold through β-augmentation. Recently, multiple lead compounds that interfere with BamA's function have been identified. We modeled and then docked one of these compounds into either the extracellular loops of BamA or the open lateral gate. With the compound docked in the loops, we found that the lateral gate remains closed during 5 μs molecular dynamics simulations. The same compound when docked in the open lateral gate stays bound to the β16 strand of BamA during the simulation, which would prevent substrate OMP folding. In addition, we simulated mutants of BamA that are resistant to one or more of the identified lead compounds. In these simulations, we observed a differing degree and/or frequency of opening of BamA’s lateral gate compared to BamA-apo, suggesting that the mutations grant resistance by altering the dynamics at the gate. We conclude that the compounds act by inhibiting BamA lateral gate opening and/or binding of substrate, thus preventing subsequent OMP folding and insertion.

## Introduction

Antibiotic resistance is a growing threat with a projected mortality rate of 10 million people per year by 2050.^1,2^ The challenging rise in antibiotic-resistant bacteria is intensified by the overuse of antibiotics in both human and animal medicine.^3−6^ Gram-negative bacteria, in particular, comprise the majority of critical and high priority pathogens responsible for the rapid rise in resistance. Gram-negative bacteria are characterized by an asymmetric outer membrane (OM), in addition to the inner membrane, and a peptidoglycan layer between them.^7−9^ The two membranes in combination with efflux pumps act as a natural mechanism of resistance to antibiotics, including hydrophobic and hydrophilic compounds.^10−13^

**Figure 1:**
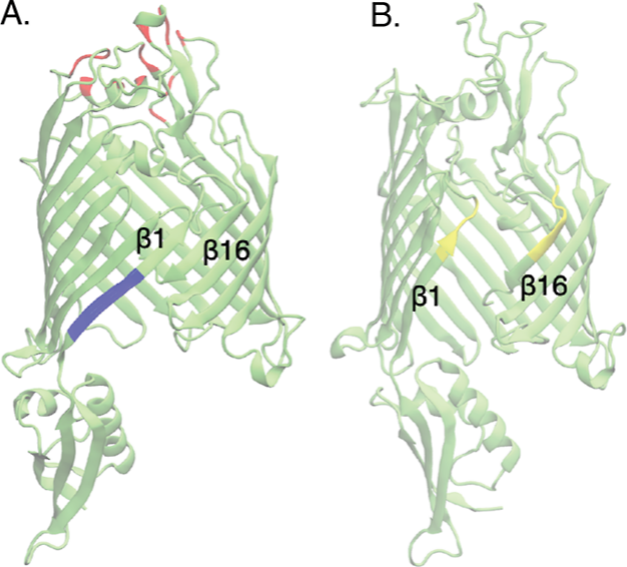
Snapshot of BamA in cartoon representation in a (A) closed lateral gate conformation and (B) open lateral gate conformation. The β1 and β16 strands forming the lateral gate are labeled in each. Red: initial binding position of CP3 in CP3-ecLs. Blue: binding position of darobactin in daro-LG. Yellow: initial binding position of CP3 in CP3-LG.

To develop novel antibiotics, the β-barrel assembly machinery (BAM) complex has recently been investigated as a potential target.^14−19^ The core component of the complex, BamA, is universal in Gram-negative bacteria, making it especially attractive.^20−23^ BamA is a sixteen-stranded β-barrel that resides in the OM.^24−26^ It possesses a lateral gate (LG) formed by β1 and β16, which has been structurally characterized in two conformations known as laterally open and closed^27−29^ (Figure 1). BamA adopts the open conformation to fold outer-membrane proteins (OMPs) and insert them laterally into the OM.^30−32^ In addition to its ubiquity, BamA is an attractive target due to its location in the OM, allowing antibiotics to avoid the permeability barriers of the membranes as well as active efflux.^23,33,34^

**Figure 2:**
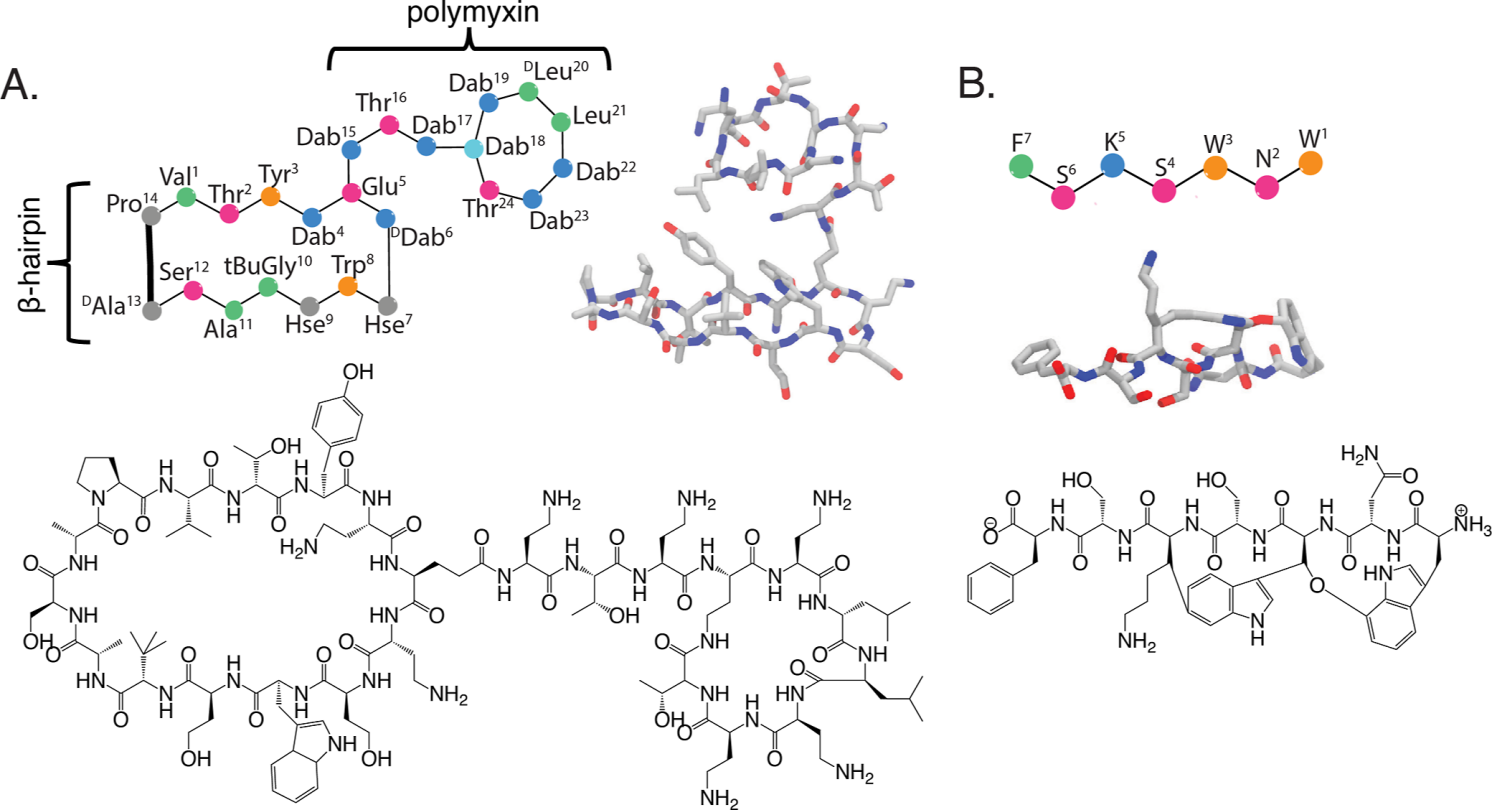
Ligands studied via MD simulations. (A) Different representations of CP3: (upper-left) cartoon, (upper-right) licorice representation without hydrogens taken from a simulation in water box, and (bottom) line structure. The β-hairpin macrocycle was optimized from murepavadin, while the circular macrocycle was optimized from polymyxin B. (B) Different representations of darobactin: (top) cartoon, (middle) licorice representation without hydrogens taken from simulation with BamA, and (bottom) line structure.

In 2019, multiple classes of compounds were identified with antibacterial activity that target BamA. Newly developed chimeric peptides from Luther et al. were derived from the combination of two existing antibiotic peptides, murepavadin and polymyxin B (Figure 2).^17^ The lead compounds were found to bind to specific residues in the extracellular loops of BamA. A second class of compounds, represented by darobactin, was first shown by Imai et al. to have direct antibacterial activity with three sets of mutations in BamA found to confer resistance.^18^ Through NMR, it was found that BamA bound to darobactin keeps the lateral gate of BamA in the closed conformation,^18^ further supported by the release of the cryo-EM structure of BamA with darobactin bound to the β1 strand.^35^

In this study, we built and simulated models of BamA and two different lead compounds. Based on the compounds from Luther et al.,^17^ we built two different systems with one of the chimeric peptidomimetic compounds docked, one in the extracellular loops of BamA and one in the open lateral gate of BamA. We also simulated the cryo-EM structure of darobactin bound at BamA’s lateral gate. In addition to ligand-bound BamA, we investigated the ramifications of identified resistance mutations on BamA structure and dynamics. We simulated three set of mutants in BamA that conferred resistance to darobactin: M1 (G429V and G807V), M2 (F394V, E435K, and G443D), and M3 (T434A, Q445P, and A705T). Based on these three mutant-BamA simulations, we identify biasing of the lateral gate toward a more open state as a likely means of resistance.

## Methods

### General System Construction

We constructed all systems with the CHARMM-GUI membrane builder.^36,37^ BamA was placed in an *Escherichia coli* asymmetric OM consisting of a phospholipid (PL) inner leaflet and a lipopolysaccharide (LPS) outer leaflet with O-antigen excluded. The protein and membrane were solvated with TIP3P water molecules, and ions were added to a concentration of 0.15 M KCl. For the LPS molecules, lipid A was neutralized with Ca^2+^, while the R1 core was neutralized with Mg^2+^. In all systems, the BamA β-barrel and POTRA5 (P5) domain were modeled; P1–P4 domains were excluded. A total of seven different systems were built (Table 1). The first two systems include a chimeric peptidomimetic ligand, denoted as CP3, bound to two different binding sites of BamA. These two systems will be denoted as CP3-ecLs and CP3-LG, respectively. The third system, labeled as daro-LG, is from a cryo-EM structure and features the ligand darobactin bound to BamA. The fourth system is BamA-apo. The next three systems are mutants of BamA that were found to be resistant to darobactin denoted as M1, M2, and M3.^18^

**Table 1:** Summary of Simulations Run in This Study^a^

#	system name	description
1	CP3-ecLs	CP3 bound to ecLs of BamA
2	CP3-LG	CP3 bound to open LG of BamA
3	daro-LG	darobactin bound to LG of BamA
4	BamA-apo	BamA β-barrel + P5 domain
5	M1—BamA G429V G807V	darobactin-resistant mutant of BamA
6	M2—BamA F394V E435K G443D	darobactin-resistant mutant of BamA
7	M3—BamA T434A Q445P A705T	darobactin-resistant mutant of BamA

a Each system was run for four ∼5 μs replicas.

### Building the Ligands

For CP3, the β-hairpin macrocycle portion murepavadin was built using the Molefacture plugin in VMD.^38^ The polymyxin B macrocycle portion of CP3 was taken from an available crystal structure (PDB ID: 5L3F).^39^ The entire CP3 was built using Psfgen in VMD, combining the murepavadin we built with the crystal structure polymyxin B. The parameter and topology files were created in part with CGenFF for missing parameters,^40^ with additional modifications made as needed. CP3 was equilibrated for 100 ns in water to ensure its stability. For darobactin, the structure of the antibiotic was part of the published cryo-EM structure (PDB ID: 7NRI), precluding the need to build it in Molefacture.^35^ The parameter and topology files were again made with CGenFF. All topology and parameter files are provided in the Supporting Information.

### Building the Simulation Systems

In total, seven different systems were simulated in this study. Two systems were built with CP3 bound to BamA. The first system, CP3-ecLs, was built with BamA in its closed conformation, with the β1 and β16 strands of the barrel anti-parallel.^41^ With BamA-closed, CP3 was docked to the extracellular loops of BamA. CP3 was docked to specific residues that were identified via NMR previously.^17^ The second system, CP3-LG, was built with BamA with an open lateral gate, taken from our previous work.^42^ This BamA originally had one β-hairpin of EspP within the open BamA lateral gate. The EspP portion was removed and instead, in the open lateral gate, the murepavadin portion of CP3 was docked. The rest of CP3 was built back in with Psfgen, resulting in BamA with an open lateral gate with CP3 docked in the space between the β1 and β16 strands. In both systems, docking of CP3 was done with HPEPDOCK with rigid body docking.^43^

In our simulations with BamA and darobactin, the protein and ligand were taken directly from the cryo-EM structure (PDB ID: 7NRI).^35^ The cryo-EM structure was taken and built into an *E. coli* OM.

### MD Protocol

Simulations were run using NAMD3^44^ (CP3-ecLs, CP3-LG, daro-LG, and BamA-apo) or Amber^45^ (M1, M2, and M3). The CHARMM36 force field for lipids^46^ and CHARMM36m for proteins^47^ were used for all. Visualization and analysis were done with VMD.^38^ All production simulations were run with hydrogen mass repartitioning (HMR) and a 4 fs timestep.^48^ Simulations were run at a constant temperature of 310 K applying Langevin dynamics and a constant pressure of 1 atm using the anisotropic Langevin piston barostat (in NAMD) or the Monte Carlo barostat (Amber). The particle mesh Ewald method^49^ was applied to evaluate the long–range interactions with a 12 Å cutoff for van der Waals interactions and a force-based switching function beginning at 10 Å.

Systems first underwent equilibration of the lipid bilayer for 0.5 ns, the protein side chains for 1 ns, and finally the entire protein for 1 ns. Unless otherwise stated, each system was then run over four replicas of 5 μs, giving a net simulation time of ∼20 μs per system.

After construction, the systems CP3-ecLs and CP3-LG were equilibrated for 200 ns with distance and hydrogen bond colvars^50^ applied to maintain the interactions between ligand and protein. The distance and hydrogen bond colvars were applied between functional groups on the ligand and residues on BamA at the interface of the ligand and protein. The colvars restraints were then released, and equilibrium simulations were run for 5 μs.

For the simulations of the BamA mutants, the lipid tails were first equilibrated for 1 ns, the entire membrane and water for 10 ns, the protein side chains for 10 ns, and finally, the whole system for 10 ns. Production simulations were then run for four replicas each of 4.7–5 μs. In total, ∼140 μs of simulations was carried out between all systems and replicas.

### Data Analysis

All analysis was performed using VMD. The hydrogen bond measurements were done with the VMD HBonds plugin with the cutoffs set at 3.5 Å for the donor–acceptor distance and 30° for the donor–acceptor hydrogen angle. For the lateral gate measurements, the number of hydrogen bonds was quantified between the backbones of BamA β1 and β16. For the simulations with the ligand bound at the LG, CP3-LG, and daro-LG, the ligand was included as part of the calculations. For CP3-LG, the ligand was selected along with the β16 strand and the hydrogen bond interactions with β1 of that entire section was calculated. Similarly, for daro-LG, the ligand was included in the selection with the β1 strand. The contact area was determined by calculating the solvent accessible surface area of the appropriate selections. For these measurements, data were collected every 10 ns. Graphs over time are shown as a running average over 25 data points.

## Results

### Interactions between Ligands and BamA

Our study focuses on two ligands that are being developed as antibiotics, CP3 (Figure 2A)^17^ and darobactin (Figure 2B).^18^ Although multiple peptidomimetics were studied, we decided to focus on CP3 due to its potency, favorable ADME properties, and extensive supporting data provided. Two models of BamA and CP3 were built. The first, referred to as CP3-ecLs, has CP3 docked to the residues on the BamA ecLs identified in NMR experiments (Figure S1A). In the second model, CP3-LG, CP3 was docked to the open lateral gate of BamA (Figure S1B). While CP3-ecLs was informed from experiments, CP3-LG was built to determine whether or not the lateral gate is a potential binding site for ligands and a strategy to prevent substrate OMP folding. Simulations of the second ligand, darobactin, started from a recent cryo-EM structure, where darobactin is bound to BamA’s β1 strand^35^ (Figure S1C).

**Figure 3:**
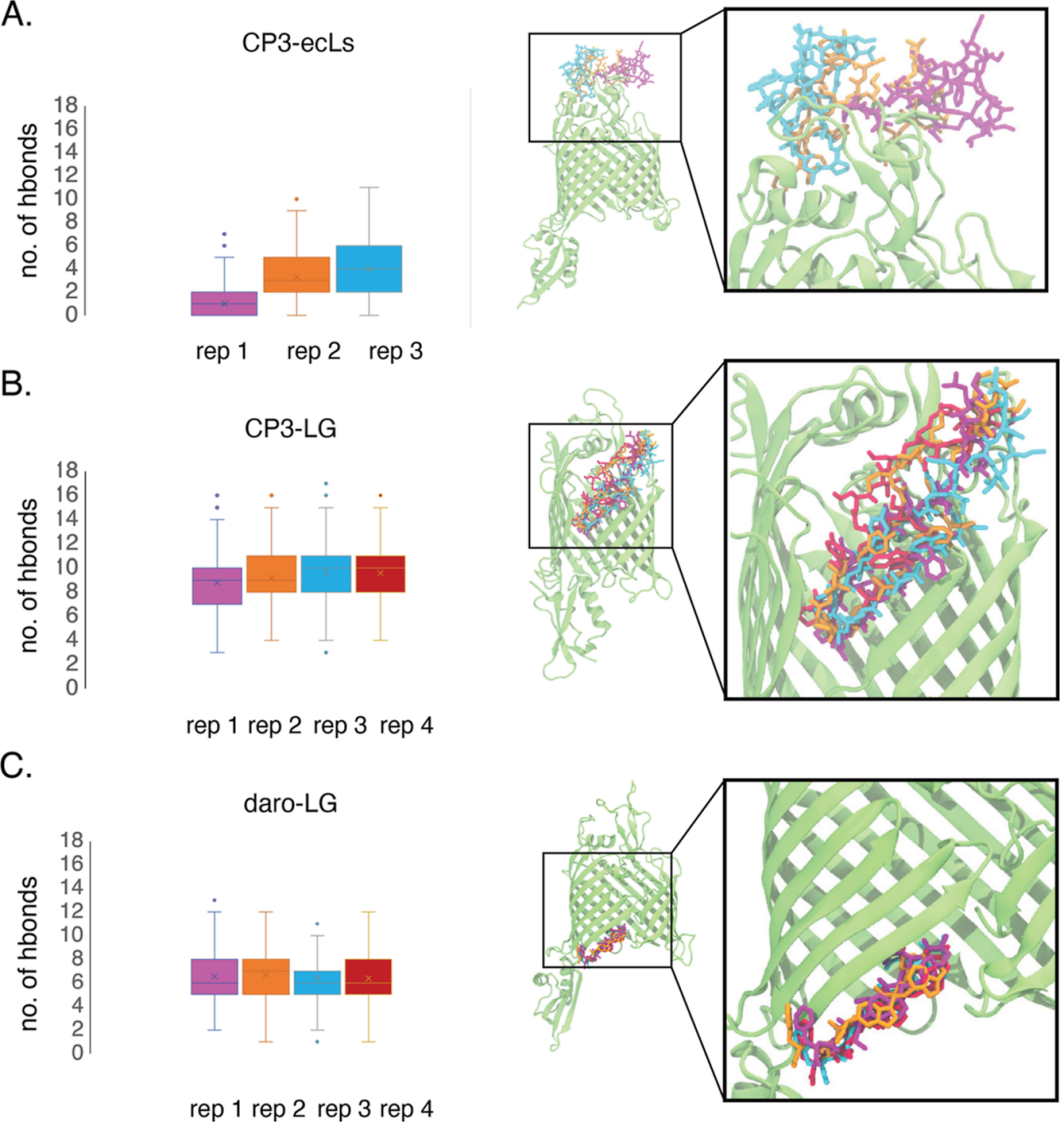
Number of hydrogen bonds measured between ligand and protein for each system represented as a box plot with standard deviation bars and outliers plotted. Adjacent to each plot is a snapshot of BamA (green) and the ligand at the end of the 5 μs simulations, colored by replica. Each snapshot is accompanied by a zoomed-in view of the binding site. (A) CP3-ecLs. (B) CP3-LG. (C) daro-LG.

Four 5 μs replica simulations of the first model of CP3-ecLs did not converge on a common binding site. However, out of the four replicas, three show CP3 remaining bound to BamA (Figure 3A). In the fourth replica, CP3 dissociated from the ecLs of BamA within the first 40 ns of the simulation. In the three replicas in which CP3 remained bound to BamA, different residues on CP3 are responsible for interactions with BamA. In particular, replicas 1 and 3 both show interactions between CP3 and BamA S752 and Q753, however via different residues on CP3 (Figure S2).

**Figure 4:**
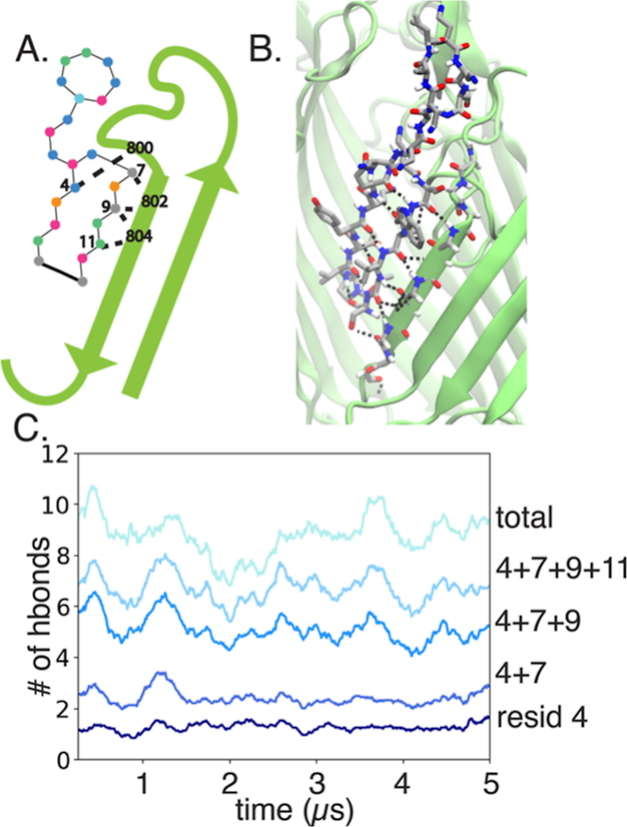
Hydrogen bond interactions for the system CP3-LG. (A) Specific hydrogen bond interactions between CP3 and BamA (residues on each labeled). (B) Snapshot of CP3-LG at the end of one of the 5 μs simulations. (C) For replica 1, number of hydrogen bonds between BamA and CP3 over time, labeled by the CP3 residue along with the total number in light blue.

In contrast to CP3-ecLs, CP3-LG shows consistent interactions maintained between ligand and protein across all four replicas. In this system, the docked structure placed CP3 next to the β16 strand of BamA. The β-hairpin portion of CP3 formed hydrogen bonds between its backbone and the backbone of BamA β16, while the macrocycle interacted with LPS of the OM (Figure S3B). Over the four 5 μs simulations, the hydrogen bonds between the backbones were maintained (Figure 3B). In particular, residues 4, 7, 9, and 11 of CP3 were responsible for consistent hydrogen bonding between CP3 and the residues E800, F802, and F804 on the BamA β16 strand (Figures 4, S4).

Together, the two models of CP3 bound to BamA lead us to hypothesize that CP3 first binds to the ecLs of BamA, interacting with both the protein and the LPS molecules through the polymyxin B macrocycle portion of the ligand. However, the interactions with the ecLs may be a transitory state for CP3 on its way to a binding pose at the lateral gate. The polymyxin B macrocycle portion of CP3 still interacts with LPS in this pose, while the β-hairpin of CP3 (optimized from murepavadin) remains bound within the open lateral gate. This possible transitory state from ecLs to another binding site in the LG is further supported by the contact area between CP3 and LPS, which is greater in CP3-LG compared to CP3-ecLs (Figure S3).

**Figure 5:**
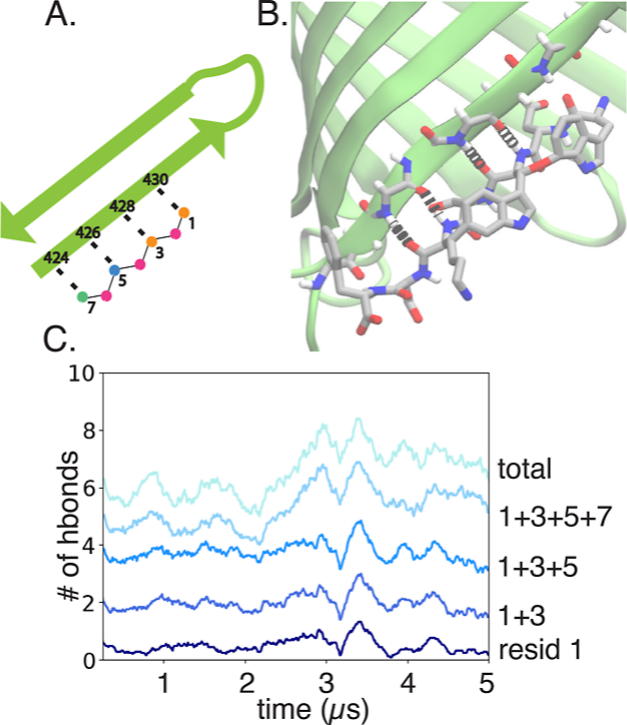
Hydrogen bond interactions for the system daro-LG. (A) Specific hydrogen bond interactions between darobactin and BamA (residues on each labeled). (B) Snapshot of daro-LG at the end of one of the 5 μs simulations. (C) For replica 1, number of hydrogen bonds between BamA and darobactin over time, labeled by the darobactin residue with the total number in light blue.

The existence of a binding site at the BamA lateral gate is complemented by the recently published cryo-EM structure of darobactin bound to BamA.^35^ In this structure, darobactin is bound to the β1 strand of BamA. In all four simulations with darobactin, the ligand remained bound at its initial position via hydrogen bonding (Figure 3C). Similar to our simulations of CP3-LG, the interactions between the peptide-like darobactin and BamA were composed of hydrogen bonds between their respective backbones, specifically between residues 1, 3, 5, and 7 of darobactin and residues G424, F426, F428, and I430 on the BamA β1 strand (Figures 5, S5).

### Characterizing the BamA Lateral Gate with and without Bound Ligands

The two ligands investigated here both showed consistent interactions with the BamA lateral gate in two of our models, CP3-LG and daro-LG, primarily through hydrogen bonding with either β1 (darobactin) or β16 (CP3). We also characterized the lateral gate’s behavior while these ligands are bound to one side of it. Previous work has shown that locking the lateral gate in a closed conformation with disulfide cross-links eliminates bacterial cell growth.^24,27^ Separation of the BamA β1 and β16 strands is one of the first steps of OMP folding in Gram-negative bacteria and is a useful metric for characterizing not only BamA itself but also the effects of the ligand on it.

**Figure 6:**
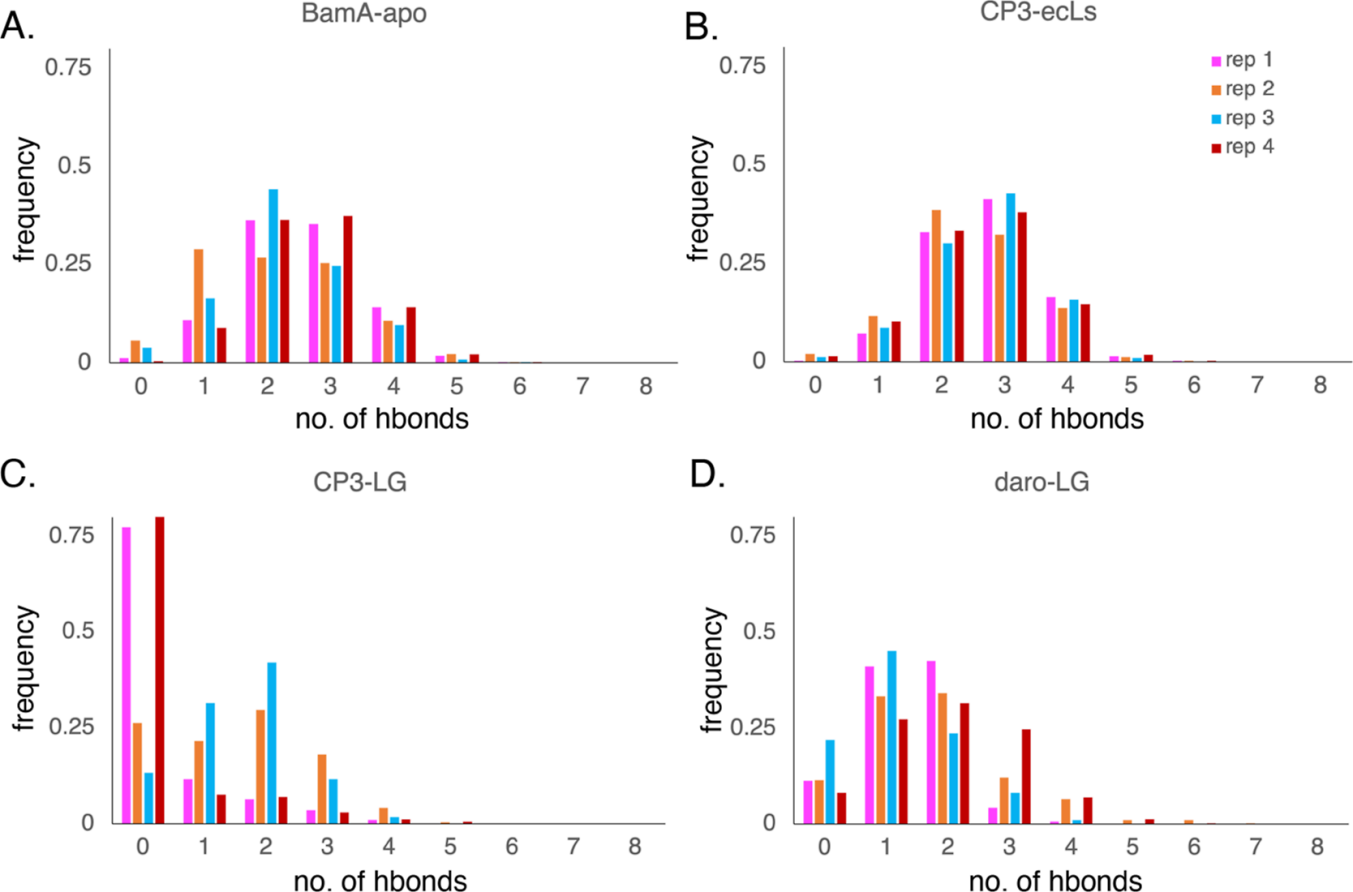
Number of hydrogen bonds between the backbones of BamA β1 and β16 strands for (A) BamA-apo, (B) CP3-ecLs, (C) daro-LG, and (D) CP3-LG. In the daro-LG and CP3-LG systems, the β1 and β16 strand selections include the ligands darobactin and CP3, respectively.

The behavior of the lateral gate is characterized here by measuring the number of hydrogen bond interactions between the β1 and β16 strands. In the first system, CP3-ecLs, the lateral gate is very similar in behavior to that of BamA-apo with typically 2–3 hydrogen bonds between gate strands, although the former (CP3-ecLs) appears to be more consistent across the four replicas (Figure 6A,B). As the ligand remains bound to the ecLs of BamA, these results suggest that it would not greatly affect the dynamics of the lateral gate.

For the simulations with CP3 bound at the open lateral gate, CP3-LG, hydrogen bonding between the two sides of the gate (β1 with CP3/β16) indicates that lateral gate dynamics have shifted toward the open state, with two of the replicas in particular having no hydrogen bonds at all for 75% of the simulations (Figure 6C). Nonetheless, the bound CP3 may still physically hinder a substrate from using much of BamA β1 for folding due to the lack of space in the lateral gate. While the gate is more weakly bound with CP3 present, CP3 stays bound to the BamA β16 strand throughout the simulation through hydrogen bonding with the backbone of BamA β16 (Figure 3B).

In the simulations with darobactin, daro-LG, the lateral gate connection is modestly weaker compared to BamA-apo, with only 1–2 hydrogen bonds typically between the two sides (β1/darobactin with β16) compared to 2–3 for BamA-apo (Figure 6A,D). However, darobactin remains bound to BamA β1 consistently throughout the duration of the simulation in all replicas (Figure 3C). Additionally, high-resolution structures of the C-terminal β-strands of OMP substrates bound to BamA reveal that the initial binding site is identical to that of darobactin,^51^ indicating that it would physically prevent a substrate OMP from utilizing BamA’s β1 strand to begin folding.

### Lateral Gate Behavior in Resistant BamA Mutants

**Figure 7:**
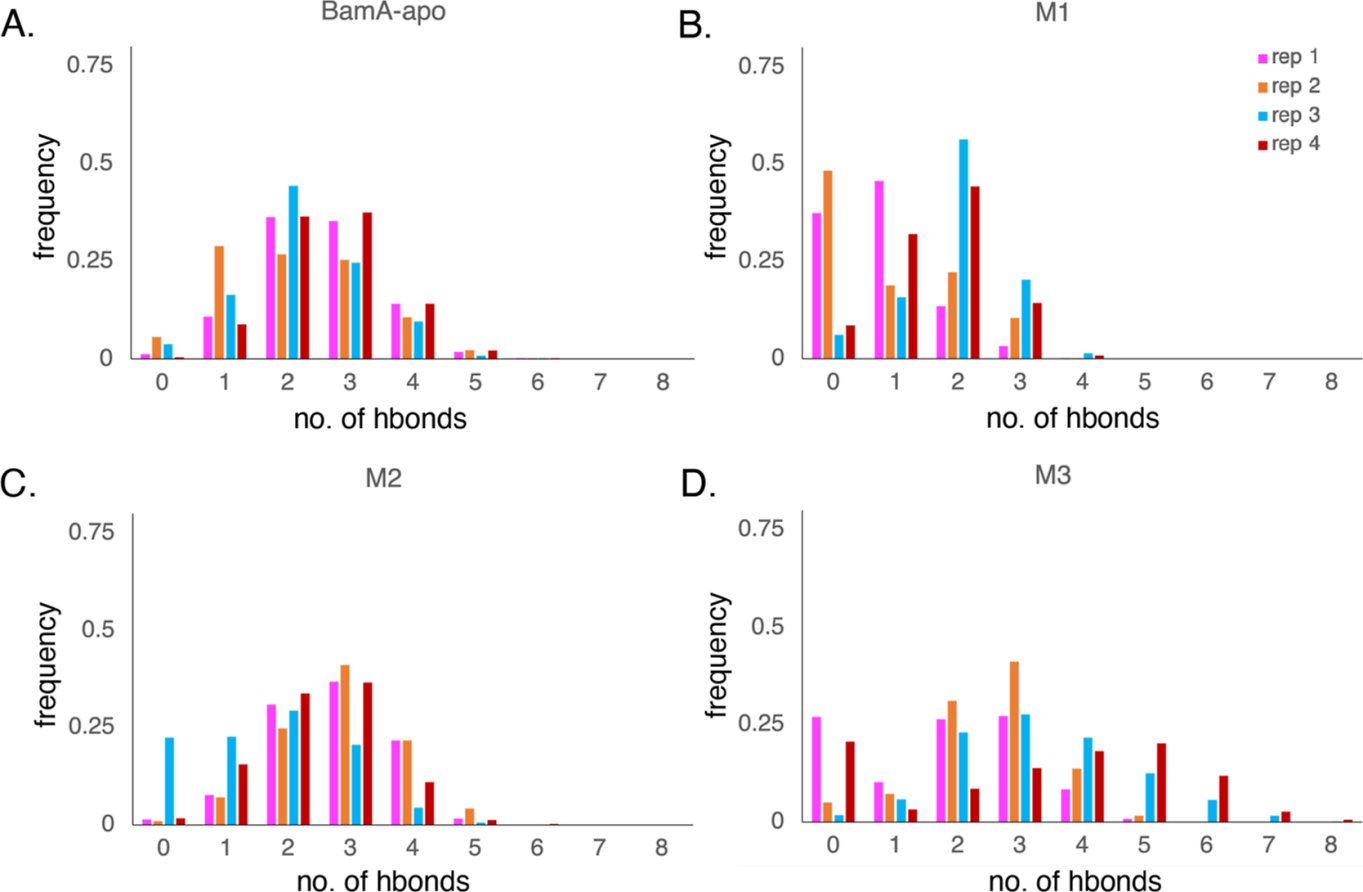
Number of hydrogen bonds between the BamA lateral gate, or the backbones of BamA β1 and β16 strands, represented by the frequency normalized over the trajectory for (A) BamA-apo, (B) M1, (C) M2, and (D) M3.

**Figure 8:**
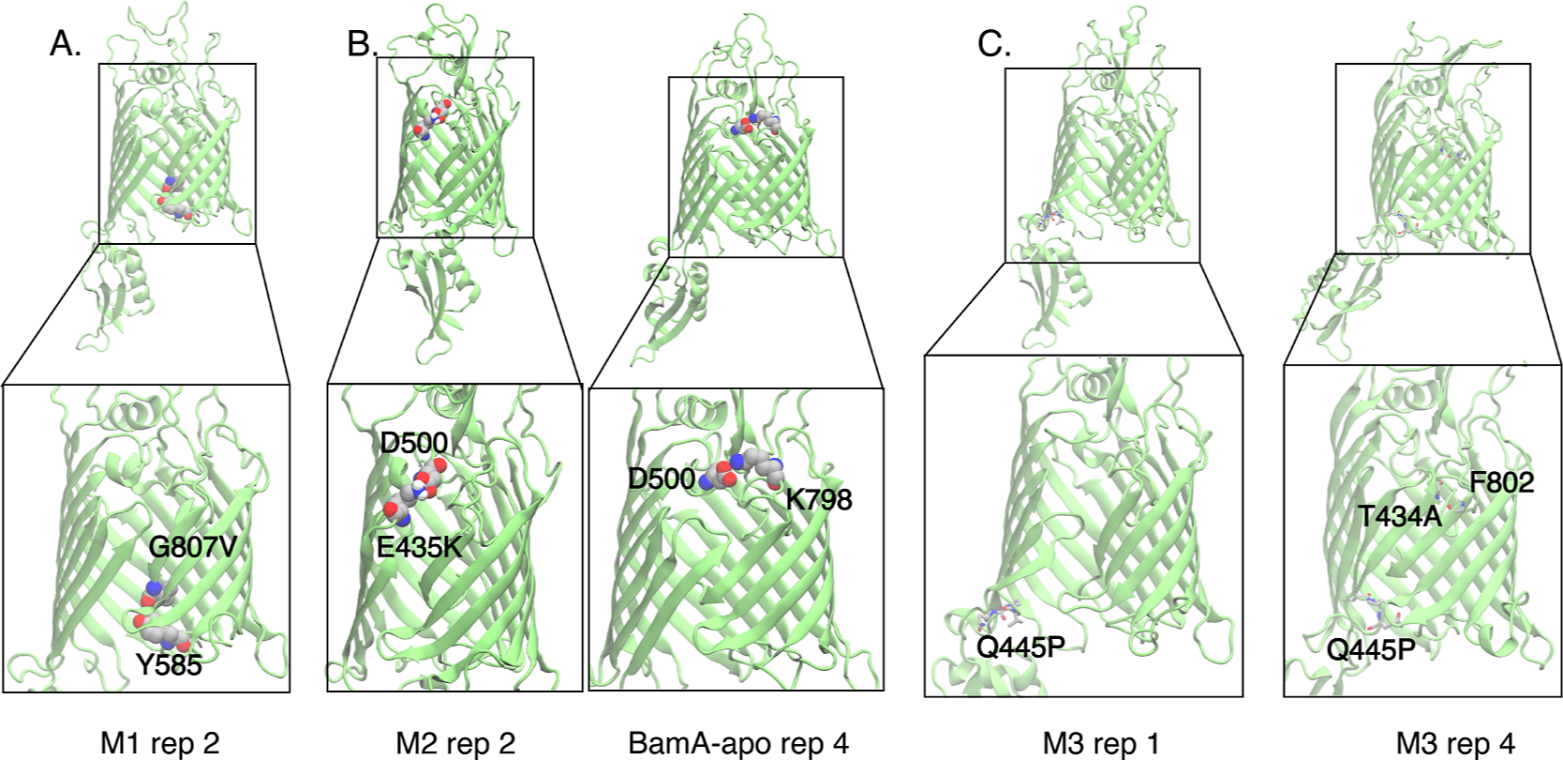
Snapshots of interesting lateral gate behavior observed in the resistant BamA mutants: (A) M1 at *t* = 3.2 μs, (B) M2 at *t* = 4.6 μs, and (C) M3 rep1 and rep4 at *t* = 5 μs.

Mutants resistant to some of the recently discovered compounds have been found,^18,19^ including three strains resistant to darobactin, each with 2–3 mutations.^18^ We modeled and simulated four replicas for each of these mutants for around 5 μs each (Figure S6). The three mutants, labeled M1 (G429V/G807V), M2 (F394V/E435K/G443D), and M3 (T434A/Q445P/A705T), display differences in behavior compared to BamA-apo, including hydrogen bonding between the lateral gate β-strands (Figure 7). Overall, M1 had fewer hydrogen bonds between BamA β1 and β16 with lateral gate behavior shifting toward the open conformation (Figure 7B). Looking more closely at the simulations, when the separation between the β-strands was the greatest, the mutated residue G807V maintained an interaction with residue Y585 (Figure 8A). This interaction pulls the β16-strand into the β-barrel cavity, separating it and creating more space between it and the β1-strand at the lateral gate. This suggests that in M1, the mutated residues affect the lateral gate dynamics, shifting it toward the open conformation.

In contrast to M1, the mutant M2 exhibited similar lateral gate behavior to BamA-apo, particularly in the hydrogen bonding (Figure 7C). However, there is one replica that exhibited a lateral gate in the open conformation more frequently. In this simulation, the mutated residue E435K, which is on ecL1, formed a salt bridge with the residue D500 on BamA’s ecL3 (Figure 8B). This salt bridge, involving two residues on the β1 side of the lateral gate, contrasts with one sometimes formed in apo-BamA between E435 and K798 on the opposite side that stabilizes the closed conformation of the gate (Figure 8B). These results indicate how resistance mutations, even when not at the lateral gate, can alter dynamics toward a more open conformation.

In the case of the mutant M3, the lateral gate dynamics are dramatically altered compared to BamA-apo. Here, the mutations are also not at the lateral gate. However, there is a mutation present on ecL1, similar to M2. In M3, both closed and open conformations of the lateral gate are exhibited within one replica, exemplifying its increased dynamics. Lateral gate behavior is not dramatically altered toward one conformation or the other, but the β1 and β16 strands have a wider possible range of hydrogen bonding along their backbones (Figure 7D). Compared to BamA-apo in which the maximum was five hydrogen bonds, a total of as many as eight hydrogen bonds were observed between the backbones of the LG in M3. When looking more closely at the simulations, it becomes apparent that the mutation Q445P has cascading effects on LG dynamics (Figure 8C). The presence of proline causes strain in the backbone of the β-strand, distorting the β2 strand. The β1 strand strains to maintain hydrogen bonding with the backbone of β2, resulting in separation between the β1 and β16 strands at the LG. The proline mutation can also result in a closed lateral gate due to a register shift at the LG. This leads to the mutated T434A residue in the BamA β1 strand to interact with F802 on the β16 strand. Overall, simulations of all three mutants suggest that altering BamA lateral-gate dynamics ameliorates the effects of darobactin.

## Discussion

In this study, two lead antibiotic compounds that target BamA, the chimeric peptidomimetic CP3 and darobactin, were simulated bound to the protein. Due to the lack of available structures of BamA bound to CP3, two different models were built and simulated with the compound docked to the ecLs (CP3-ecLs) or the open lateral gate (CP3-LG). For darobactin, the cryo-EM structure in which the ligand is bound to the BamA β1 strand was simulated. In most of these simulations, the BamA lateral gate displayed distinct behaviors compared to the apo system. In addition to the simulations with ligand bound, three mutants of BamA resistant to darobactin were modeled and simulated.

The first model of CP3 docked to BamA’s ecLs was created based on NMR spectroscopy suggesting them as a binding site.^17^ As CP3 contains a β-hairpin peptide macrocycle, we speculated that it could also bind to the lateral gate of BamA; docking of CP3 to the open gate produced a model in which CP3 was stably bound to BamA’s β16. Both docking sites, ecLs and lateral gate, proved reasonably stable over the course of 5 μs simulations, with dissociation occurring in only one CP3-ecLs replica (Figure 3). Based on the two different models of CP3 bound to BamA, we hypothesize that the ligand first binds to the ecLs of BamA via interactions between the polymyxin moiety of CP3 and LPS molecules in the OM, after which it may, at least under some circumstances, move to bind to the open lateral gate of BamA, supported by the similarity between the β-hairpin macrocycle of CP3 and the natural substrate of BamA. CP3 can also permeabilize the OM,^17^ suggesting that the ecLs of BamA are not its only binding site.

The lateral gate as a potent binding site for ligands targeting BamA is further supported by the discovery of darobactin. The cryo-EM structure revealed darobactin bound to β1 of BamA, and simulations here show that binding site to be stable (Figure 5). Darobactin bound to the lateral gate prevents substrate folding by blocking the initial binding site.^51^ Furthermore, it affects lateral gate behavior, weakening the connection between the two sides (Figure 6). A recent study using EPR spectroscopy distance measurements between BamA ecLs concluded that binding of another darobactin variant, darobactin-B, biases BamA toward a closed state;^52^ when we measure the distance between the same residues (L501 and S755), we do not see an increase in daro-LG simulations compared to BamA-apo, in agreement (Figure S7). The darobactin-resistant mutants also show altered lateral gate dynamics, either in terms of the number of hydrogen bonds at the gate (Figure 7) or the specific interactions across it (Figure 8). These results suggest that biasing BamA toward a more dynamic and/or laterally open state compensates for the substrate blockage created by darobactin.

The mechanism of OMP insertion via BamA has recently converged on the hybrid-barrel model. In this model, lateral gate opening is among the first steps in order to expose the BamA β1 and β16 strands, allowing the substrate OMP to fold within the open lateral gate.^27,28,53−56^ Multiple cryo-EM structures of BamA with a substrate have been resolved recently, always displaying a hybrid barrel irrespective of the stage of OMP insertion.^30−32,57^ The ligands investigated in this study affect BamA and its lateral gate dynamics by staying bound at either the β1 or β16 strands. The resistant mutants also tend to alter lateral gate dynamics. Together, the data suggest that further development of antibiotics targeting BamA should focus on its lateral gate, albeit noting the potential for resistant mutants to rapidly arise.

## Supporting Information Available

Initial poses used for simulations of ligand-bound BamA; for CP3-ecLs, snapshots of the interactions between ligand and BamA for replica 1 and replica 3; contact area between ligand and LPS (upper leaflet of the outer membrane) for CP3-ecLs and CP3-LG; number of hydrogen bonds between ligand and protein for the system CP3-LG, quantified per residue for rep 2, rep 3, and rep 4; number of hydrogen bonds between ligand and protein for the system daro-LG, quantified per residue for rep 2, rep 3, and rep 4; three resistant mutants of BamA studied with mutated residues in the van der Waals representation: BamA-apo, M1 - G429V G807V, M2 - F394V E435K G443D, and M3 - T434A Q445P A705T; and distance between the residues 501 and 755 on BamA extracellular loops for the simulations BamA-apo and daro-LG (PDF)
Topology and parameter files for CP3 and darobactin and VMD scripts to build them (ZIP)
