# Supplementary material for: Drug Binding to BamA Targets Its Lateral Gate
Source: J Phys Chem B. 2023 Aug 17;127(34):7509–17. doi: 10.1021/acs.jpcb.3c04501 (PMC10476194; doi:10.1021/acs.jpcb.3c04501)
Supplement: Supplementary file 1 — jp3c04501_si_001.pdf [file jp3c04501_si_001.pdf]

Supporting Information: Drug Binding to BamA Targets Its Lateral Gate

Katie M. Kuo,[†] Jinchan Liu,[‡] Anna Pavlova,[¶] and James C. Gumbart[*,¶,†]

†*School of Chemistry and Biochemistry, Georgia Institute of Technology, Atlanta, GA 30332, United States*

‡*Department of Molecular Biophysics and Biochemistry (MB&B), Yale University, New Haven, CT 06510, United States*

¶*School of Physics, Georgia Institute of Technology, Atlanta, GA 30332, United States*

E-mail: gumbart@physics.gatech.edu

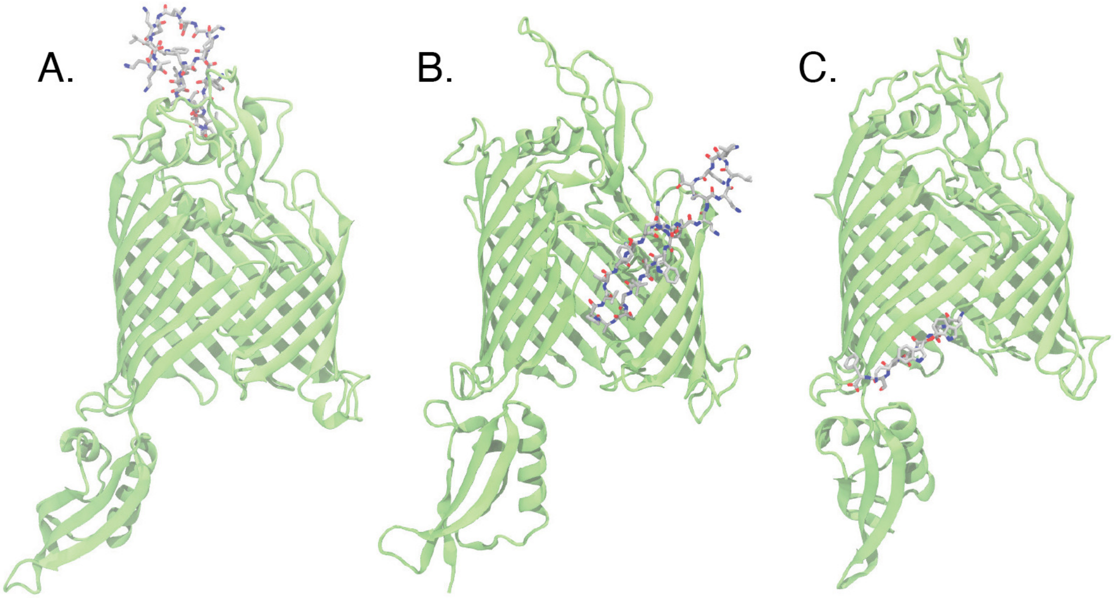

Figure S1: Initial poses used for simulations of ligand-bound BamA (green). A) CP3-ecLs B) CP3-LG C) daro-LG.

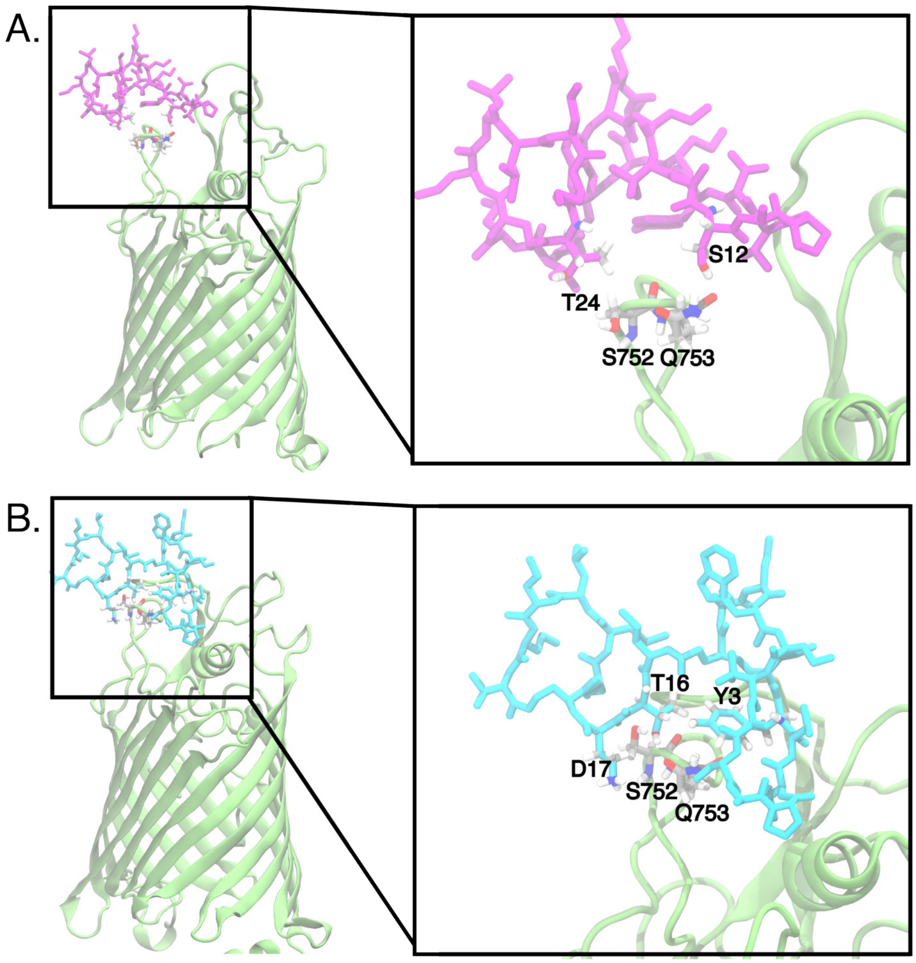

Figure S2: For CP3-ecLs, snapshots of the interactions between ligand and BamA for A) replica 1 and B) replica 3.

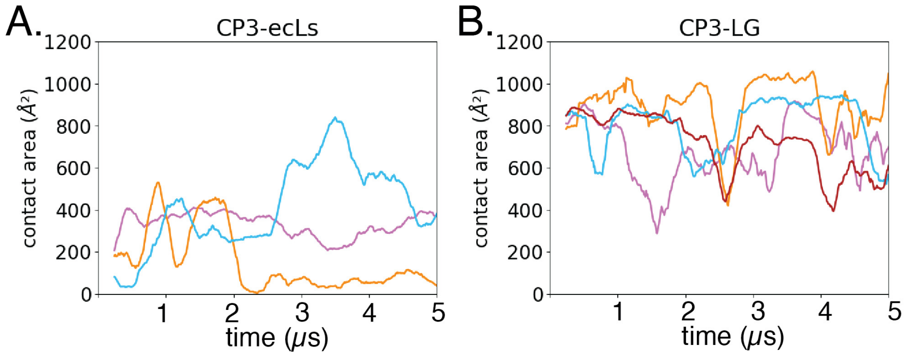

Figure S3: Contact area between ligand and LPS (upper leaflet of the outer membrane) for A) CP3-ecLs and B) CP3-LG.

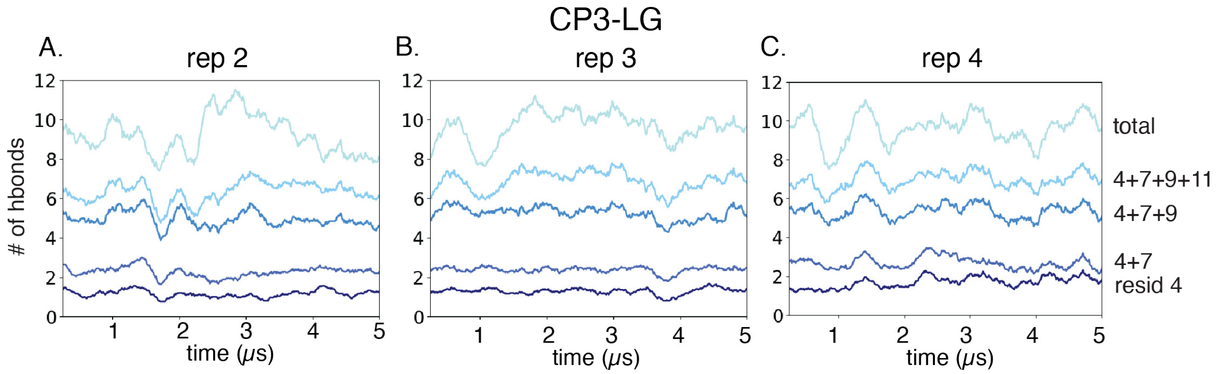

Figure S4: Number of hydrogen bonds between ligand and protein for the system CP3-LG, quantified per residue for A) rep 2, B) rep 3, and C) rep 4.

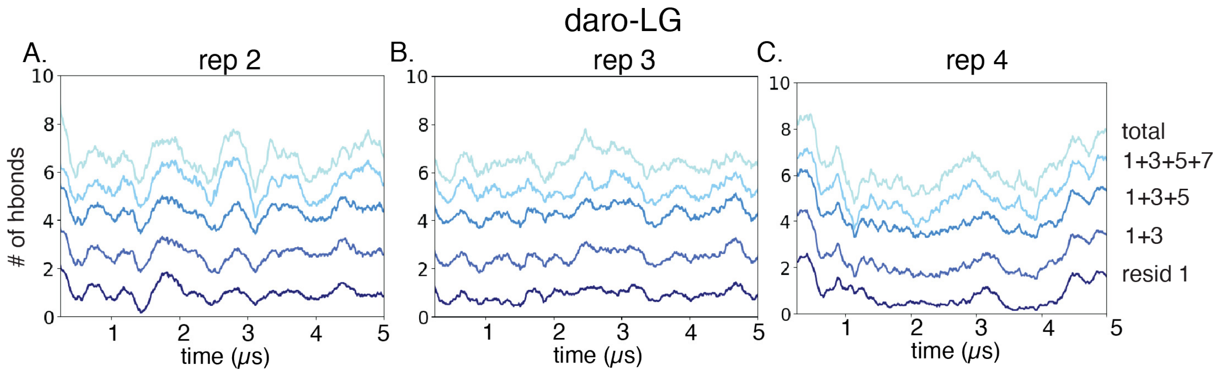

Figure S5: Number of hydrogen bonds between ligand and protein for the system daro-LG, quantified per residue for A) rep 2, B) rep 3, and C) rep 4.

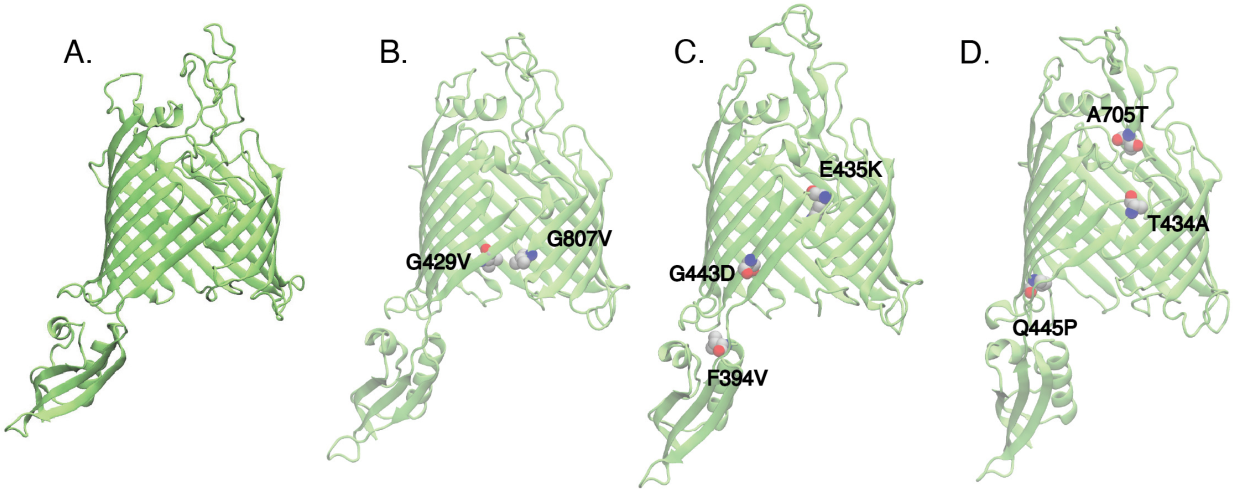

Figure S6: The three resistant mutants of BamA studied with mutated residues in the van der Waals representation: A) BamA-apo, B) M1 - G429V G807V, C) M2 - F394V E435K G443D, and D) M3 - T434A Q445P A705T.

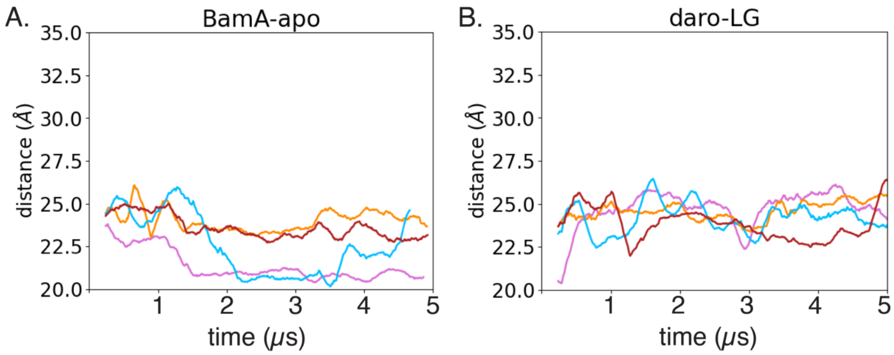

Figure S7: The distance between the residues 501 and 755 on BamA extracellular loops for the simulations A) BamA-apo and B) daro-LG.
